# Attrition of T-Cell Functions and Simultaneous Upregulation of Inhibitory Markers Correspond with the Waning of BCG-Induced Protection against Tuberculosis in Mice

**DOI:** 10.1371/journal.pone.0113951

**Published:** 2014-11-24

**Authors:** Subhadra Nandakumar, Sunil Kannanganat, James E. Posey, Rama Rao Amara, Suraj B. Sable

**Affiliations:** 1 Division of Tuberculosis Elimination, Centers for Disease Control and Prevention, Atlanta, Georgia, United States of America; 2 Department of Microbiology and Immunology, Yerkes National Primate Research Center, Emory University, Atlanta, Georgia, United States of America; University of Cape Town, South Africa

## Abstract

*Mycobacterium bovis* bacille Calmette-Guérin (BCG) is the most widely used live attenuated vaccine. However, the correlates of protection and waning of its immunity against tuberculosis is poorly understood. In this study, we correlated the longitudinal changes in the magnitude and functional quality of CD4^+^ and CD8^+^ T-cell response over a period of two years after mucosal or parenteral BCG vaccination with the strength of protection against *Mycobacterium tuberculosis* in mice. The BCG vaccination-induced CD4^+^ and CD8^+^ T cells exhibited comparable response kinetics but distinct functional attributes in-terms of IFN-γ, IL-2 and TNF-α co-production and CD62L memory marker expression. Despite a near life-long BCG persistence and the induction of enduring CD4^+^ T-cell responses characterized by IFN-γ and/or TNF-α production with comparable protection, the protective efficacy waned regardless of the route of vaccination. The progressive decline in the multifactorial functional abilities of CD4^+^ and CD8^+^ T cells in-terms of type-1 cytokine production, proliferation and cytolytic potential corresponded with the waning of protection against *M. tuberculosis* infection. In addition, simultaneous increase in the dysfunctional and terminally-differentiated T cells expressing CTLA-4, KLRG-1 and IL-10 during the contraction phase of BCG-induced response coincided with the loss of protection. Our results question the empirical development of BCG-booster vaccines and emphasize the pursuit of strategies that maintain superior T-cell functional capacity. Furthermore, our results underscore the importance of understanding the comprehensive functional dynamics of antigen-specific T-cell responses in addition to cytokine polyfunctionality in BCG-vaccinated hosts while optimizing novel vaccination strategies against tuberculosis.

## Introduction

Tuberculosis (TB) is the most devastating bacterial disease of all time and is responsible for over 1.3 million deaths annually [1]. The only vaccine available against TB is *Mycobacterium bovis* Bacille Calmette-Guérin (BCG),6 an attenuated strain of *M. bovis*. It is the most widely used vaccine in human history, and is recommended in >157 countries [2]. BCG efficiently prevents severe forms of childhood TB [3], [4], but has highly variable efficacy against pulmonary TB in adolescents and adults [5], [6]. Therefore, extensive research efforts are ongoing to develop new TB vaccines with superior protective efficacy. Since over 4 billion individuals have received BCG to date, it is generally accepted that the development of new vaccination strategies should consider the extensive BCG-vaccination coverage [7]–[9]. Accordingly, future vaccination against TB would involve priming with BCG or an improved live mycobacterial vaccine followed by a boost with subunit vaccine [9], [10]. A standard approach for screening new vaccine candidates or strategies to improve BCG-induced immunity starts with the evaluation of immunogenicity and protective efficacy in animal models of TB, in particular, the mouse model. However, despite being at the center of new TB vaccination strategies and its extensive use as a reference standard in many preclinical and clinical vaccine trials, our knowledge of the protective immune responses induced by a near century old BCG vaccine is limited in mice and humans.

Although it is known that BCG induces durable immunity in the mouse model [11],[12], understanding of the kinetics of immune response in-terms of expansion, peak and contraction of the T-cell and antibody response is incomplete, and the optimal time to boost BCG-immunity is uncertain. These gaps have hampered the development of effective prime-boost vaccination regimens using BCG. As boosting T-cell responses during the expansion phase may lead to T-cell exhaustion and activation-induced cell death [13], [14], boosting would be most desirable after the effector phase of BCG-primed responses and when the memory T-cell population is established [15]. We reasoned that the evaluation of growth and persistence of BCG after different routes of administration in mice and the simultaneous investigation of the kinetics of the magnitude and functional quality of T-cell responses generated, together with the longitudinal changes in the protective efficacy against *Mycobacterium tuberculosis* (*Mtb*) challenge, may provide an important insight into the correlates of protection and factors associated with the waning of BCG-immunity.

The BCG vaccine is most commonly administered to newborns by the intradermal route. A recent clinical trial in infants has, however, demonstrated equivalent protective efficacy using intradermal and percutaneous vaccination routes [16]. Furthermore, TB is primarily a respiratory disease and it has been hypothesized that vaccination directed at the respiratory mucosa may provide the best opportunity for protection against infection with *Mtb*. The animal experimental evidence suggests that local immune defense mechanisms might be crucial for the protective immunity against TB, for example, those stimulated by mucosal immunization via the lower respiratory tract [17], [18]. Therefore, we investigated the dynamics of BCG immunity traversing the entire life-span of mice following intranasal (i.n., mucosal) and subcutaneous (s.c., parenteral) vaccination.

Our results showed that BCG-induced CD4^+^ and CD8^+^ T-cell responses and protection peak between 12–32 weeks but wanes significantly by 78 weeks regardless of i.n. or s.c. vaccination. The magnitude of CD4^+^ and CD8^+^ T cells with sound multi-effector capacity in-terms of type-1 cytokine production, proliferation and cytolytic potential correlated best with protection against *Mtb*. Notably, increase in the dysfunctional and terminally-differentiated T cells coincided with the waning of BCG-induced protection. Our results provide previously undocumented important insight into how BCG persistence and the immune senescence affect T-cell functionality in mice and highlight the need to develop vaccination strategies that prevent T-cell attrition and exhaustion.

## Results

### BCG persists and induces a durable cell-mediated immune response in mice

**Figure 1:**
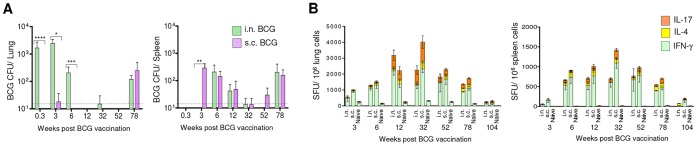
BCG persists and induces a durable cell-mediated immunity in mice. (A–B) BALB/c mice were vaccinated with 1×10^6^ CFU of BCG Copenhagen by intranasal (i.n.) or subcutaneous (s.c.) route. At 7 different time points after vaccination at the same time, mice were euthanized and their lungs and spleen were isolated. (A) BCG load in the two organs of individual mice was determined. Detection limit of the assay as shown by dotted line was 30 CFU/organ, and data shown are mean CFU ± s.e.m. in the lung (left) and spleen (right) from two independent experiments using 5 individually analyzed mice per group at each time point. The experiment was not performed at the104-week time point. (*P<0.05, ** *P*<0.01, *** *P*<0.001, **** *P*<0.0001 using 1-way analysis of variance (ANOVA) with Tukey's post-test). (B) In parallel, pooled cells from the lung (left) or spleen (right) of BCG-vaccinated and naïve mice (*n* = 4/time point/group) were stimulated with WCL in a cultured ELISPOT assay and IFN-γ, IL-4 and IL-17A SFU were enumerated. Data are means ± s.e.m. of individual cytokine SFU constituting the total cytokine responses of 2 (at week 3, 78 and 104), 3 (at week 32) or 4 (at week 6 and 12) independent experiments evaluated in triplicate. Responses are significant (*P*<0.001) in vaccinated compared to naïve mice at 6 time points after week 3 using 1-way ANOVA with Tukey's post-test.

To understand the growth and persistence of BCG after mucosal or parenteral vaccination, BALB/c mice were vaccinated with 10^6^ BCG Copenhagen colony forming units (CFU) by i.n. and s.c. route, and BCG titers were determined in the lung and spleen at 7 different time points. BCG vaccination by the i.n. route resulted in a high bacillary load in the lung. About 0.2–0.5% of the total BCG administered following i.n. vaccination was detected in the lung at 48 h, while BCG could not be detected in the lung at 48 h after s.c. vaccination and only a low numbers of bacilli (45±14 CFU) were detected in the lung at week 3 (Fig. 1A). In the spleen, peak BCG load was found at week 3 after s.c. vaccination while it took 6 weeks for BCG to be detected following i.n. vaccination. The BCG CFUs declined by week 12 to 32 but persisted at very low or undetectable levels until week 52. However, by week 78, BCG burden resurged in the two organs of both vaccinated groups. These results demonstrate the chronic persistence of BCG in mice.

In parallel, we determined the magnitude and kinetics of BCG-induced cell-mediated immune response using *Mtb* whole-cell lysate (WCL) for *in vitro* stimulations of lung and spleen cells. The frequencies of WCL-specific IFN-γ, IL-17 and IL-4 spot-forming units (SFU) were measured using a cultured ELISPOT assay. Although i.n. vaccination-induced total cytokine SFU peaked earlier in the lungs (i.e., at week 12) than those produced by s.c. vaccination (which peaked at week 32), the magnitude of WCL-specific total cytokine response in two organs was statistically comparable between the routes of vaccination when the total SFU at 7 different time points were compared (Figure 1B). The total cytokine response by either route was dominated by higher frequencies and proportions of IFN-γ SFU, although at the peak of response the frequencies of IL-17 SFU were significantly greater compared to early (6 week) and late (78 week) time points in the lung. These results suggest that though BCG vaccination induced type-1 immune response declines with age, the nature of response continues to be predominantly type-1 after i.n. or s.c vaccination

When the antibody response was investigated in the sera of two vaccinated groups, we observed similar kinetics for WCL-specific IgG-antibody response by ELISA, but it was significant only in the sera of s.c. BCG-vaccinated mice (Fig. S1A). The WCL-specific IgG response was characterized by greater proportions of IgG2a and IgG2b subclass antibodies (Fig. S1B). Overall, these results demonstrate that i.n. and s.c. BCG vaccination induces a strong cell-mediated response following early bacillary load and is maintained for more than 8 months. The peak of immune response in the lung and spleen coincides with decrease in the BCG burden and persistence of bacilli at very low levels.

### BCG vaccination-induced CD4^+^ and CD8^+^T cells exhibit distinct cytokine profile

**Figure 2:**
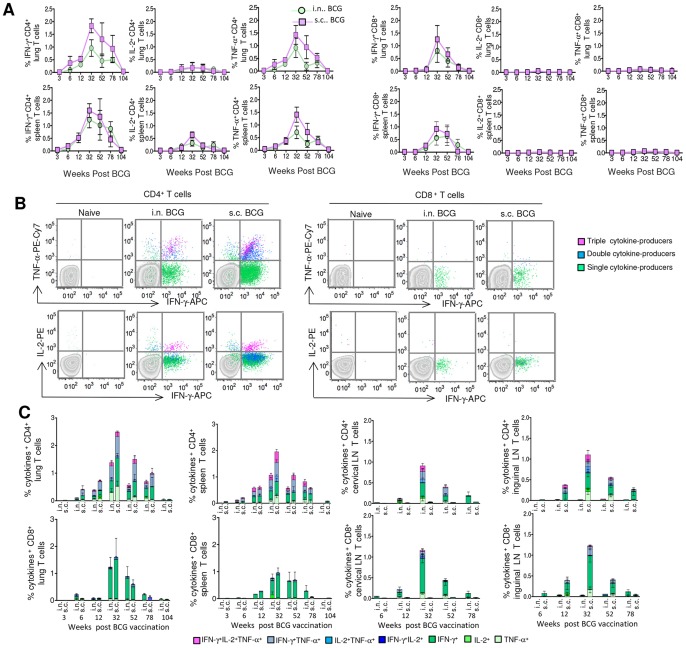
BCG vaccination induces polyfunctional CD4^+^ but monofunctional CD8^+^ T-cell response. (A–C) The lung and spleen cells of i.n. and s.c. BCG-vaccinated mice (*n* = 4/time point/group) were stimulated with WCL, and the magnitudes and polyfunctionality of CD3^+^CD4^+^ and CD3^+^CD8^+^ T cells in-terms of IFN-γ, IL-2 and TNF-α production were determined using polychromatic flow cytometry. (A) The longitudinal changes in the magnitudes of WCL-specific individual IFN-γ, IL-2 or TNF-α-producing cells among CD4^+^ or CD8^+^ T cells in the lung and spleen are plotted. (B) Representative dot plots show the frequency of WCL-specific IFN-γ and IL-2 or IFN-γ and TNF-α-producing cells among splenic CD4^+^ and CD8^+^ T cells from one mouse per vaccinated group at week 32 in comparison with age-matched naïve control, and the distributions of single, double and triple-cytokine-producers are illustrated. (C) The magnitudes of 7 possible combinations of WCL-specific cytokine-producing subsets constituting total cytokine^+^ CD4^+^ or CD8^+^ T cells in the lung, spleen, cervical lymph node (CLN) and inguinal lymph node (ILN) are depicted. The data (A, C) are mean ± s.e.m. responses of 2 (at week 3, 78 and 104), 3 (at week 32) or 4 (at week 6 and 12) independent experiments.

To understand the temporal changes in the magnitude and quality of BCG-induced CD4^+^ and CD8^+^ T-cell responses, we evaluated the frequencies of WCL- and short term culture filtrate (STCF)-specific IFN-γ, IL-2 and TNF-α-producing CD4^+^ and CD8^+^ T cells in the lung, spleen, draining and distant lymph nodes (LNs) by polychromatic flow cytometry. The WCL and STCF represent a complete repertoire of *Mtb* antigens as opposed to individual purified antigens or cocktails of few immunodominant antigens used for *in vitro* stimulations. We found comparable magnitudes of WCL-specific cytokine-producing CD4^+^ T cells between i.n. and s.c. BCG-vaccinated groups (Fig. 2A), when the frequencies of IFN-γ, IL-2, or TNF-α-expressing CD4^+^ T cells were analyzed individually in the lung and spleen. The magnitudes of WCL-specific cytokine-producing CD8^+^ T cells were also comparable between the two vaccinated groups. The T-cell responses peaked at week 32 in the lung and spleen and gradually waned thereafter, with similar expansion and contraction trend in the two BCG-vaccinated groups.

When the polyfunctionality of WCL-specific T cells was analyzed in-terms of co-expression of IFN-γ, TNF-α and IL-2 by individual cells [19], [20], we found that the CD8^+^ T-cell response in all organs investigated was dominated almost exclusively by IFN-γ single-producers (Fig. 2B and 2C). In contrast, CD4^+^ T cells were polyfunctional at the peak, and the response was dominated by higher magnitudes of IFN-γ and TNF-α single- and double-positive cells. These results demonstrate that BCG vaccination generates CD4^+^ and CD8^+^ T-cell responses of distinct quality. Contrary to the belief that BCG vaccination induces a weak CD8^+^ T-cell response [21], these data demonstrate the significant presence of IFN-γ-producing CD8^+^ T cells at peak time points. Although the percentages of WCL-specific total cytokine-producing cells among CD4^+^ and CD8^+^ T cells were comparable, the frequencies of CD4^+^ T cells in the T lymphocytes (CD3^+^) of the organs investigated were approximately three-fold higher than the frequencies of CD8^+^ T cells, thereby confirming the dominance of CD4^+^ T-cell response after BCG vaccination during the entire response kinetics in mice.

The BCG-induced early T-cell responses tend to be strongest in the compartments most proximal to the site of vaccine administration [22], and it is believed that the distribution of specific T cells may become uniform in the local and distant compartments as the vaccination-induced immune response matures. Therefore, we investigated the specific T-cell responses simultaneously in the draining and distant LNs after i.n. and s.c. BCG vaccination at 5 different time points. Vaccination by the i.n. route induced higher WCL-specific cytokine-producing CD4^+^ and CD8^+^ T cells in the draining superficial cervical lymph nodes (CLNs), while s.c. vaccination induced more T-cell responses in the inguinal lymph nodes (ILNs) which drains the flank (the site of s.c. vaccination) (Fig. 2C). Responses in the draining CLNs of i.n.-vaccinated and ILNs of s.c.-vaccinated group also peaked at week 32, but, significantly higher magnitudes (*p*<0.001) of WCL-specific T cells were observed only in the corresponding draining LNs as compared to the distant LNs at all 5 time points investigated (Fig. 2C). These results demonstrate a near lifelong compartmentalization of T-cell responses in the draining LNs depending on the route of BCG vaccination.

Furthermore, the mean frequencies of WCL-specific T cells waned after week 32 in the two vaccinated groups with significantly higher polyfunctional CD4^+^ T cells at week 32 (*p*<0.05) compared to week 78 in the three organs investigated, demonstrating decline in the magnitude and quality of BCG vaccination-induced T-cell response. By week 104, the magnitude of WCL-specific cytokine-expressing CD4^+^ and CD8^+^ T cells had waned to levels similar to those observed at week 3 (Fig. 2C), and we observed similar T-cell cytokine response dynamics using *Mtb* STCF (Fig. S2A and S2B). However, in contrast, we did not find any significant difference in the magnitude of WCL-specific cytokine-expressing T cells in young (8-week-old) and old (104-week-old) mice, 6 weeks after BCG vaccination (Fig. S2C), indicating that the decline in the immune response with time after BCG vaccination was not merely due to age-related senescence. Together, these data demonstrate the gradual waning of BCG-induced CD4^+^ and CD8^+^ T-cell responses after 32 weeks of vaccination, regardless of the route of administration.

### BCG-induced CD4^+^ and CD8^+^ T cells express cytolytic potential that wanes with time

**Figure 3:**
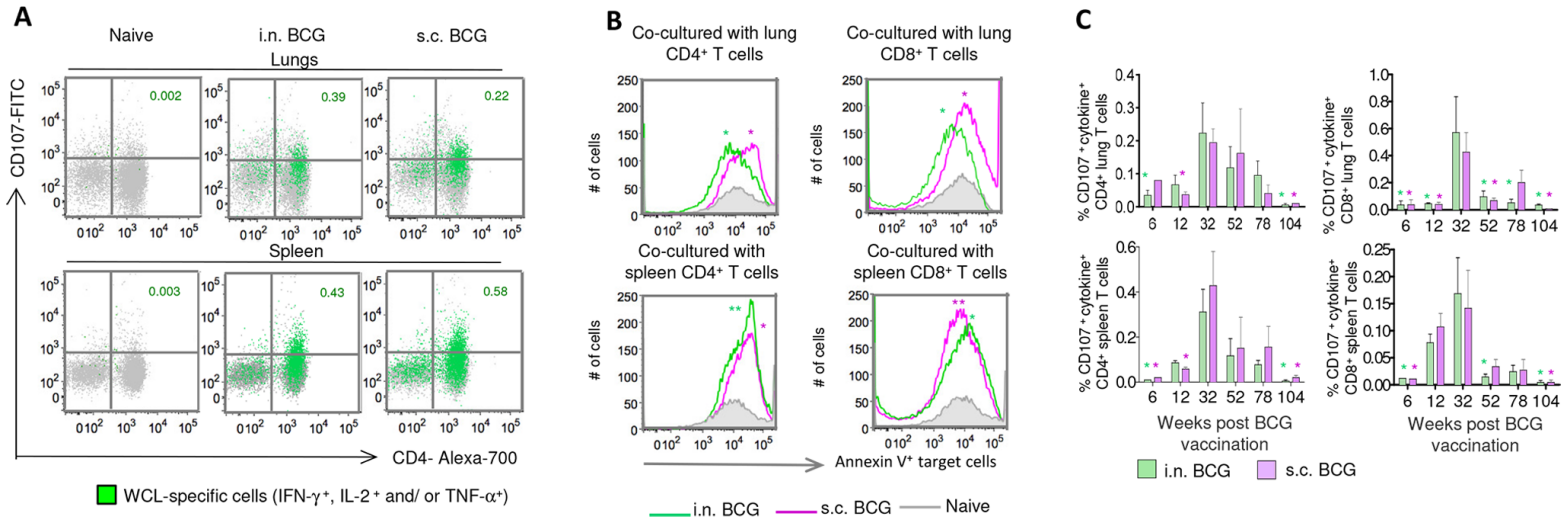
BCG vaccination-induced CD4^+^ and CD8^+^ T cells express cytolytic potentials. (A–B) The lung and spleen cells from i.n. or s.c. BCG-vaccinated mice were stimulated with WCL after staining with CD107a and CD107b and the CD107 a/b expression was evaluated on T cells (gated on CD3^+^ cells). (A) The CD107a/b expression on CD4^+^ T cells of lung and spleen of one representative mouse from the control and vaccinated groups at week 32 is shown. The green dots represent the WCL-specific IFN-γ, IL-2 and/or TNF-α-producing cells. (B) Representative histograms show the annexin-V expression on the cell surface of WCL-pulsed target cells (i.e., BMDCs gated on CD3^−^CD11c^+^ cells) after co-culture with the purified CD4^+^ (left) or CD8^+^ (right) T cells (at 1∶20 ratio) from the pooled lungs (upper panel) or spleens (lower panel) of BCG-vaccinated or age-matched naïve mice (*n* = 3/group). (*P<0.05, ** *P*<0.01, using 1-way ANOVA with Tukey's post-test and by comparing frequencies of annexin-V^+^ BMDCs (CD3^−^CD11c^+^ cells) of vaccinated mice and naive controls). (C) The summary of temporal changes in the magnitudes (%) of CD107 a/b upregulation on CD4^+^ (left) or CD8^+^ (right) T cells expressing IFN-γ, IL-2 and/or TNF-α after WCL-stimulation. The data are mean ± s.e.m. responses of 2 (at week 3, 6, 78 and 104) or 3 (at week 32 and 12) independent experiments (*n* = 4/time point/group). (*P<0.05, ** *P*<0.01, using 1-way ANOVA with Tukey's post-test, depicting significantly less responses compared to corresponding 32-week time points).

To further characterize the temporal changes in the quality of BCG-induced T cells, we assessed the degranulation potential of CD4^+^ and CD8^+^ T cells, measured as the surface mobilization of CD107a and CD107b [19], [23], [24]. The WCL-specific CD4^+^ and CD8^+^ T cells in the lung and spleen of BCG-vaccinated mice expressed CD107 (Fig. 3A), and the route of administration did not affect their degranulation potential. Furthermore, both CD4^+^ and CD8^+^ T cells purified from the two organs of vaccinated groups induced cytolysis in target cells based on the up-regulation of apoptosis marker annexin-V on the surface of WCL-pulsed bone-marrow-derived dendritic cells (Fig. 3B), lung macrophages (Fig. S3A), or peritoneal macrophages (Fig. S3B) upon co-culture. The degranulation potential of both T-cell subsets peaked at week 32, and the percentages of WCL-specific CD107-expressing T cells declined significantly by week 78 (*p*<0.05 for CD4^+^ and *p*<0.01 for CD8^+^ T cells) in BCG-vaccinated mice (Fig. 3C).

### BCG-induced CD4^+^ and CD8^+^ T cells exhibit distinct memory phenotype in-terms of CD62L expression and their proliferative potential decreases during waning

**Figure 4:**
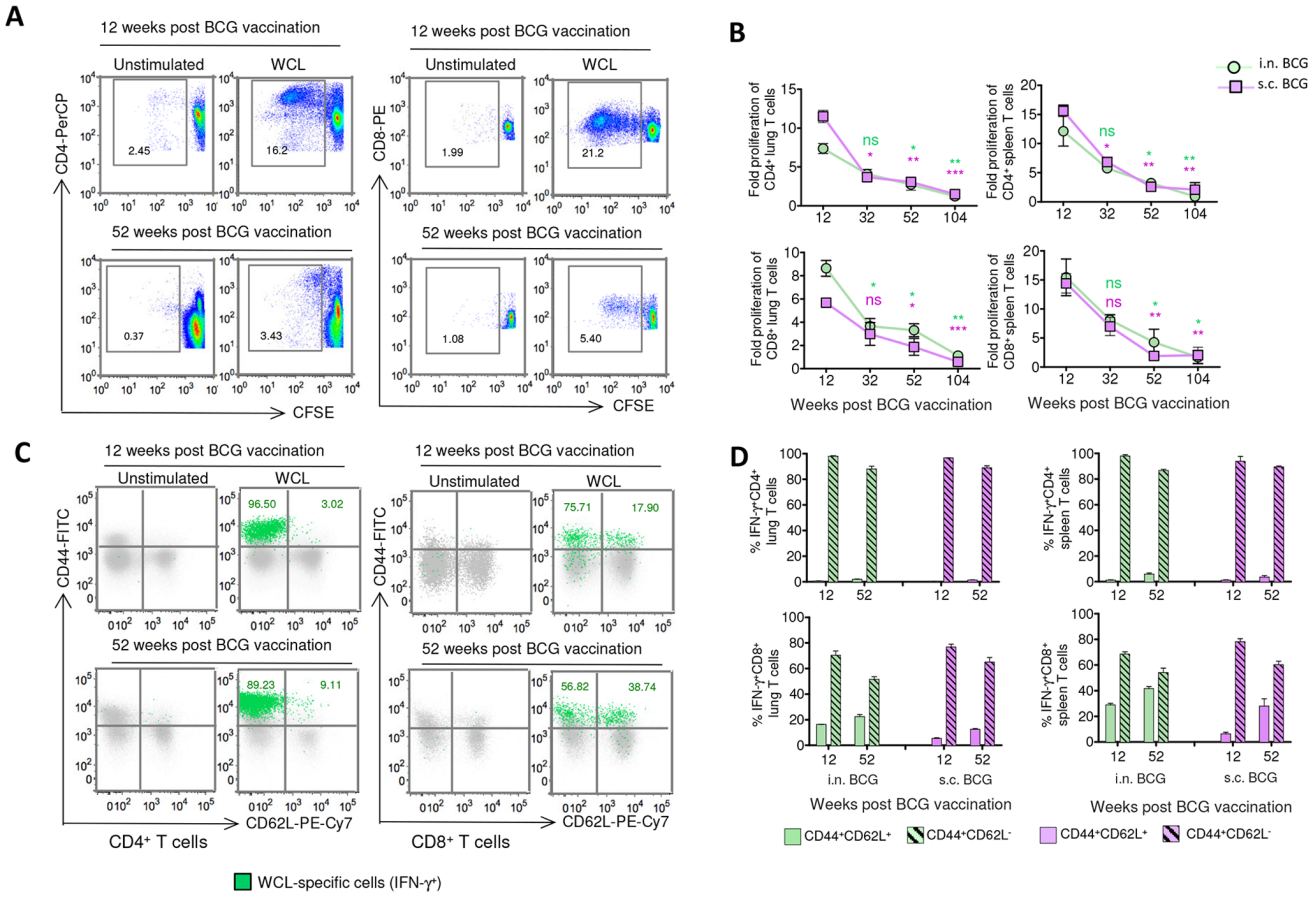
BCG-induced T cells predominantly exhibit effector or effector memory phenotype and their proliferative potential wanes with time. (A–B) The proliferation of CD4^+^ and CD8^+^ T cells from the lung and spleen of the BCG-vaccinated mice was investigated based on CFSE-dilution at 72 h following WCL or no antigen stimulation. (A) Representative plots illustrate the proliferation of pooled lung CD4^+^ and CD8^+^ T cells of vaccinated mice (*n* = 4) from one experiment at the expansion and contraction phase. (B) The longitudinal changes in the proliferative potential of the lung and spleen CD4^+^ or CD8^+^ T cells from the two vaccinated groups at 4 different time points were summarized and are presented as the fold increase in proliferation compared to unstimulated controls (*P<0.05, **P<0.01 using 1-way ANOVA with Tukey's post-test compared to corresponding week 12). (C) The CD44 and CD62L expression on the pooled splenic CD4^+^ and CD8^+^ T cells of vaccinated mice (*n* = 4) from one representative experiment at the expansion and contraction phase are shown. The green dots represent WCL-specific IFN-γ-producing T cells. (D) The magnitudes of WCL-specific IFN-γ-producing CD44^+^CD62L^+^ and CD44^+^CD62L^−^ CD4^+^ and CD8^+^ T cells from the lung and spleen of two groups at week 12 and 52. The data in (B) and (D) are mean ± s.e.m. responses of 2 independent experiments using pooled organ cells (*n* = 4 mice/time point/group).

Next, we assessed the association between contracting T-cell responses and other functional abilities such as proliferative potential and up-regulation of memory markers by T cells. Using CFSE-dilution assay, we found the highest WCL-specific proliferative ability of CD4^+^ and CD8^+^ T cells *in vitro* at the expansion phase (week 12) after i.n. or s.c. vaccination (Fig. 4A and 4B). Thereafter, the proliferation decreased at week 32 and further waned significantly by week 52.

Simultaneously, we investigated the memory phenotype by assessing the expression of CD44 and CD62L or CCR7 [25], and found that >90% of lung and spleen WCL-specific IFN-γ^+^CD4^+^ T cells expressed CD44^+^CD62L^−^ activated effector or effector memory (T_EM_) phenotype at week 12 and 52 (Fig. 4C and 4D). Higher percentages (22.45–41.60%) of WCL-specific IFN-γ^+^CD8^+^ T cells expressed CD44^+^CD62^+^ central memory (T_CM_) phenotype (Fig. 4D), but, both CD4^+^ and CD8^+^ T cells did not produce significant IL-2 or express another classical T_CM_ marker, CCR-7, during the contraction phase (Fig. S4A–C). These results suggest generation of predominantly effector/T_EM_ responses but weak T_CM_ responses following BCG vaccination.

### T-cell dysfunction markers are up-regulated during waning of BCG responses

**Figure 5:**
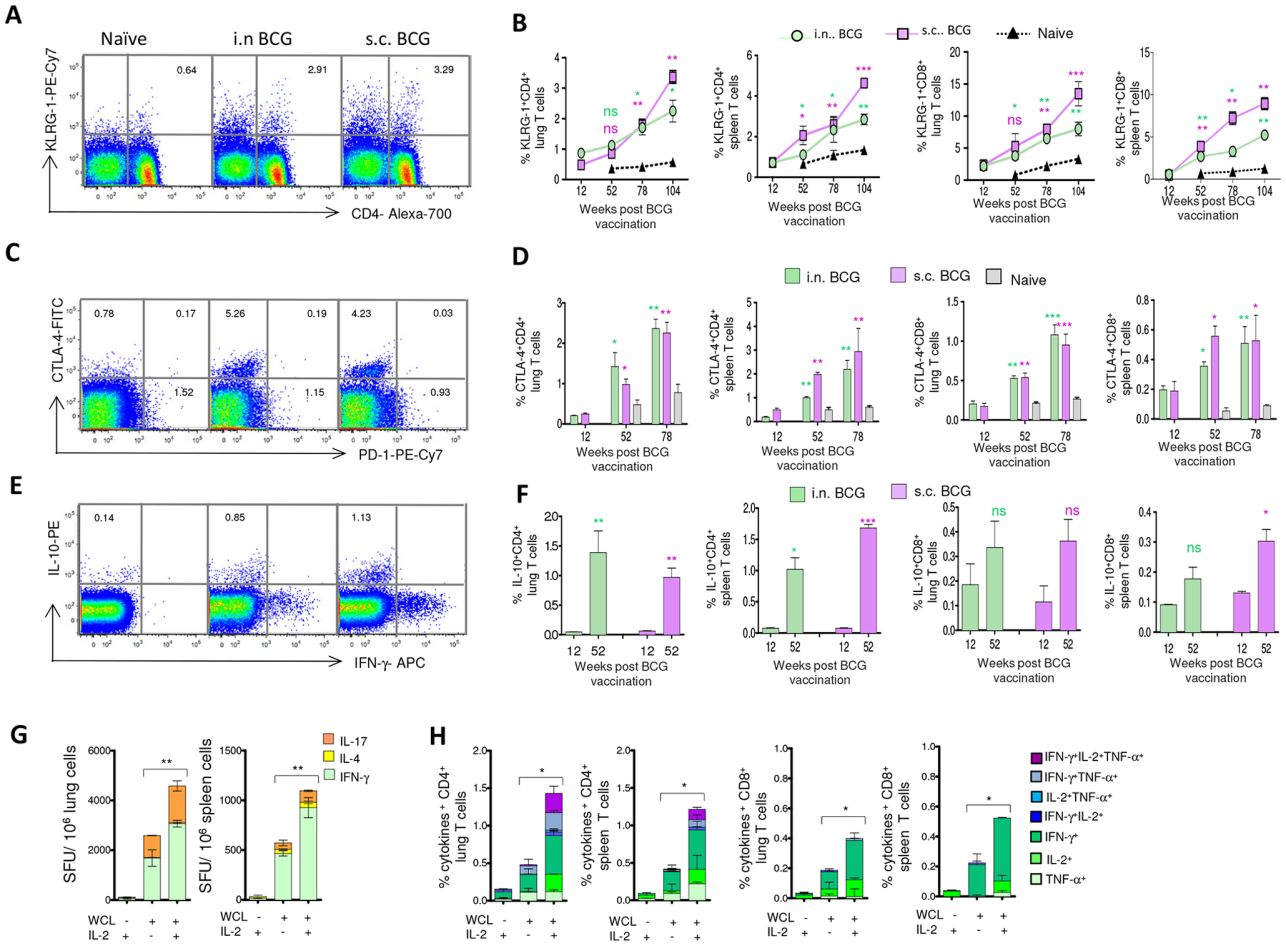
T-cell dysfunction markers are up-regulated during waning of BCG responses. (A) The expression of KLRG-1 on the pooled spleen T cells of age-matched naïve, i.n. or s.c. BCG-vaccinated mice (*n* = 4) at week 78. (B) Longitudinal changes in the magnitude of KLRG-1^+^ CD4^+^ and CD8^+^ T cells from the lung and spleen. (C) CTLA-4 and PD-1 expression on the pooled splenic CD4^+^ T cells of vaccinated or age-matched naive mice (*n* = 4) at week 78. (D) Summary of magnitude of CTLA-4^+^ CD4^+^ and CD8^+^ T cells in the lung and spleen. (E) The WCL-specific IL-10 production by the pooled splenic CD4^+^ T cells of vaccinated or age-matched naïve mice. (F) Summary of magnitude of WCL-specific IL-10^+^ CD4^+^ and CD8^+^ T cells in the lung and spleen. (G–H) The spleen and lung cells isolated from i.n. BCG-vaccinated mice at week 78 were treated with rIL-2 to evaluate potential to reinvigorate WCL-specific CD4^+^ and CD8^+^ T-cell responses. (G) The WCL-specific IFN-γ, IL-4 and IL-17 SFU after treatment with or without rIL-2. (H) The magnitude of WCL-specific cytokine-producing 7 subsets of CD4^+^ and CD8^+^ T cells by flow cytometry. The data in figure B, D, F are mean ± s.e.m. responses of 2 independent experiments using pooled organ cells (*n* = 4 mice/time point/group). * *P*<0.05, ** *P*<0.01 compared to the corresponding 12-week time point by 1-way ANOVA with Tukey's post-test. 5G, H are mean ± s.e.m responses of pooled cells evaluated in triplicates. *Significant using 1-way ANOVA with Tukey's post-test.

Chronic infections often lead to impaired T-cell responses characterized by a progressive loss of T-cell functions, terminal differentiation and eventual T-cell exhaustion [26]–[29]. Since BCG chronically persists in mice, we sought to determine whether up-regulation of T-cell inhibitory receptors and markers of T-cell dysfunction corresponded with the contraction of BCG-induced T-cell response and simultaneous decrease in T-cell functionality. We investigated the surface expression of cytotoxic T-lymphocyte antigen 4 (CTLA-4), programmed cell death protein 1 (PD-1), and killer cell lectin-like receptor subfamily G member 1 (KLRG-1), and the intracellular production of IL-10 by T cells. The KLRG-1 and CTLA-4 expression on CD4^+^ and CD8^+^ T cells of vaccinated mice increased significantly by week 78 compared to week 12, and compared to age-matched na?ve mice (*p*<0.05) (Fig. 5A–5D), suggesting that these changes are not simply age related [30]. Following WCL stimulation, >97% of CTLA-4^+^ or KLRG-1^+^ T-cell subsets did not produce IFN-γ, IL-2 or TNF-α (data not shown). However, we did not observe any significant increase in the percentages of PD-1-expressing CD4^+^ or CD8^+^ T cells during the contraction phase in BCG-vaccinated mice (Fig. 5C). Apart from the inhibitory-receptor signaling, cytokines like IL-10 also exert suppressive effect and mycobacterial reactivation [31], [32], and we found increased WCL-specific IL-10-producing CD4^+^ and CD8^+^ T cells at week 52 as compared to week 12 in BCG-vaccinated mice (Fig. 5E and 5F). Collectively, these results suggest that the waning of BCG-induced T-cell responses corresponds with the increased frequency of inhibitory markers-expressing CD4^+^ and CD8^+^ T cells.

Of importance, *in vitro* rIL-2 treatment of the lung and spleen cells significantly increased WCL-specific IFN-γ and IL-17 SFU and the total cytokine (IFN-γ, IL-2 or TNF-α) response at week 78 (Fig. 5G and 5H), indicating that the dysfunctional T-cell responses could be partly rescued by IL-2 therapy.

### Waning of BCG-induced protection against *Mtb* coincides with decreased T-cell functionality

**Figure 6:**
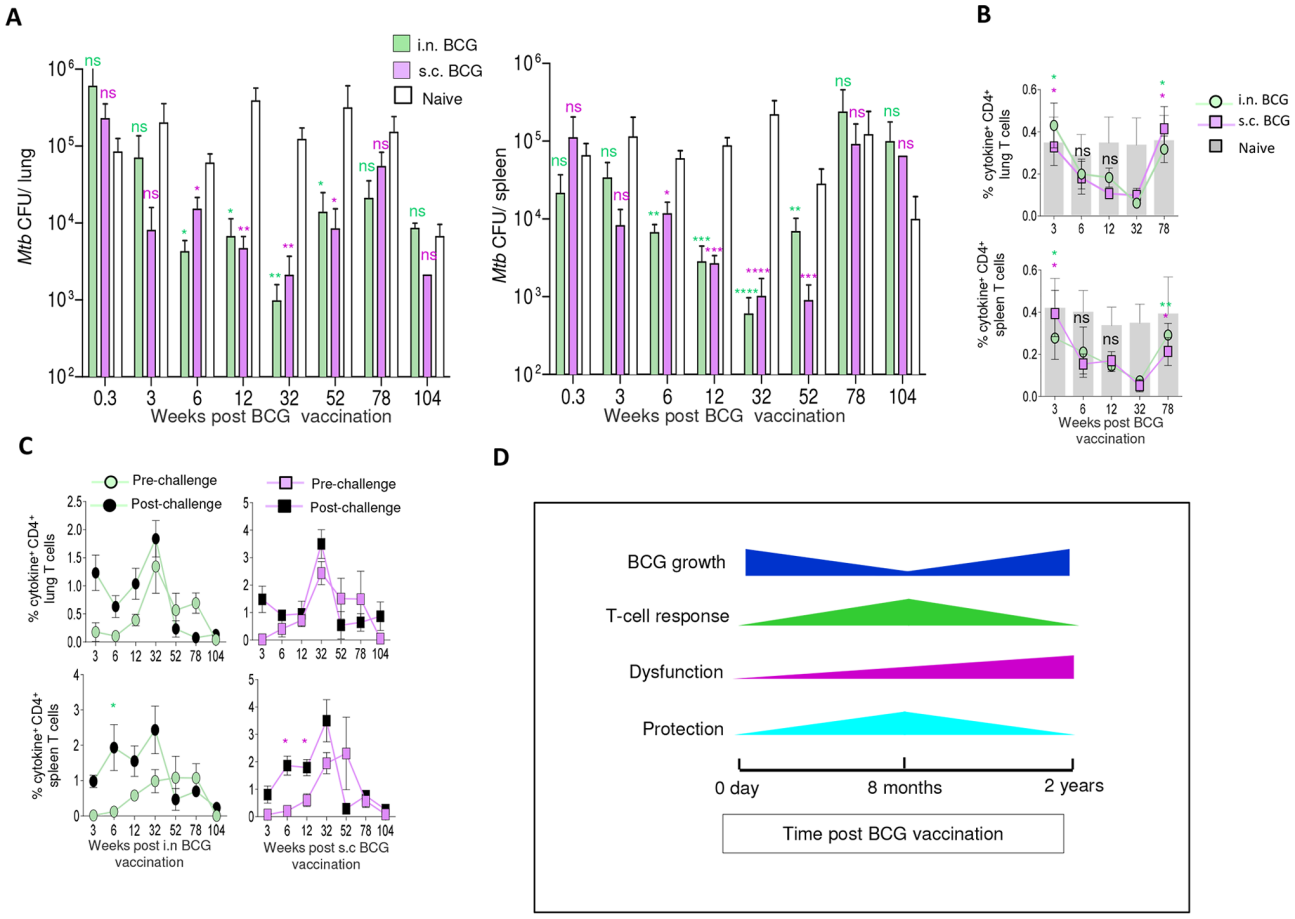
Waning of BCG-induced protection against *Mtb* coincides with decreased T-cell functional capacity. (A–C) BCG-vaccinated or age-matched naïve mice were infected with *Mtb* Erdman, and 6 weeks later the spleen and lungs were isolated. (A) Differences in *Mtb* load between vaccinated and control mice are shown. The data are mean ± s.e.m. *Mtb* CFU in each organ at eight different time points involving two to four independent experiments each using five individually analyzed mice per time point. (*P<0.05, ** *P*<0.01, *** *P*<0.001, compared to the corresponding age-matched naïve control by Kruskal-Wallis test followed by Dunn's post-test). (B) The longitudinal changes in the frequency of rESAT-6+rCFP-10-specific total CD4^+^ cytokine^+^ (of IFN-γ, IL-2 and TNF-α) T cells from the lung (upper panel) and spleen (lower panel) of vaccinated and naïve mice 6 weeks after challenge (*P<0.05, ** *P*<0.01 compared to the corresponding week 32 BCG-vaccinated mice by 1-way ANOVA and Tukey's post-test). At the 32-week time point, rESAT-6+rCFP-10-specific responses in naïve mice are statistically higher compared to the corresponding vaccinated groups (*P*<0.05). (C) The magnitudes of WCL-specific total CD4^+^ cytokine^+^ T cells before and 6 weeks after *Mtb* challenge. The data are mean ± s.e.m. responses measured by intracellular cytokine staining (ICS) of four mice per time point. (*P<0.05 comparing pre- and post-challenge WCL-specific responses using 1-way ANOVA and Tukey's post-test) (D) The schematic summary of longitudinal changes in BCG load in the lung and spleen of i.n. and s.c. BCG-vaccinated mice over a period of 2 years and its correlation with the magnitude and functional capacity of T cells and levels of protection against *Mtb* infection.

To understand the kinetics and durability of BCG-induced protection, we challenged i.n. and s.c. BCG-vaccinated mice with *Mtb* Erdman at different time points after vaccination, and *Mtb* load was determined in the lung and spleen 6 weeks later. Our data demonstrate that the route of vaccination did not significantly alter the protective efficacy against *Mtb* at all eight infection time points investigated compared to age-matched na?ve controls (Fig. 6A). The protection was found between 6–52 weeks with maximum efficacy at week 32 (log_10_ CFU reduction 1.77–2.1 in lung and 2.34–2.56 in spleen), but eventually waned by week 78. Previously elderly (2-year-old) mice have been shown to effectively control early *Mtb* infection [33], and in our model, we consistently found low CFU burden in the lung or spleen of 104-week-old elderly na?ve mice compared to simultaneously infected 12-week-old young control mice 6 weeks after challenge (data not shown), confirming that the low CFU levels in week-104 groups (Fig. 6A) were not due to failed infections. The frequency of ESAT-6+CFP-10-specific cytokine-producing T cells in the lung and spleen evaluated as a surrogate of *Mtb* load after challenge [34] correlated inversely with the degree of protection (Fig. 6B and S5A), with significantly more ESAT-6+CFP-10-specific CD4^+^ T cells in week-3 and -78 as compared to week-32 groups, 6 weeks post-challenge. Furthermore, *Mtb* challenge during the expansion phase of BCG-induced response resulted in a better recall of WCL-specific T-cell responses in the two organs (Fig. 6C and S5B). Conversely, challenge during the contraction phase demonstrated decreased frequency of WCL-specific cytokine-producing CD4^+^ and CD8^+^ T cells compared to the levels observed pre-challenge. Together, these results suggest that the degree of BCG-induced protection correlates with the magnitude and intact functional capability of specific CD4^+^ and CD8^+^ T cells (Fig. 6D).

## Discussion

We found increased BCG load in the lung as compared to spleen following i.n. vaccination while s.c. vaccination resulted in relatively higher levels in the spleen as compared to lung by 3 weeks. Similar results have been reported earlier after i.n. vaccination [35], and it is known that following s.c. vaccination initial BCG multiplication occurs in the draining ILN and results in higher bacterial dissemination to the spleen than the lung by week 4 [36]. It has been suggested that BCG chronically persists in the draining lymph nodes of mice after s.c. vaccination [12], and may serve as a reservoir for dissemination to the lung and spleen. In our study BCG sporadically disseminated to the lung in low numbers during early and late time points after s.c. vaccination. Previous studies of the shorter duration have suggested that BCG never or only rarely disseminates to the lung after s.c. vaccination [37], while others have reported that BCG disseminates to the lung in low numbers during a 20-week study [36]. In view of the recent report of bone marrow stem cells as a niche for dormant *Mtb*, further studies are required to characterize reservoirs of persistent BCG in mice [38]. Despite a significant decline in the BCG-burden by week 12 and the persistence of bacteria in low numbers thereafter at week 32–52, BCG-induced *Mtb*-specific T-cell responses and the degree of protection against subsequent *Mtb* challenge peaked at week 12–32 in the lung and spleen in our study. This occurred regardless of the early differences in the tissue localization of BCG-burden and the pronounced differences in the location of specific T cells in the draining LNs depending on the route of vaccination. We did not find any significant difference in the strength of protection against *Mtb* challenge between i.n. and s.c. BCG vaccination at all-time points investigated using a moderate vaccine dose. The dose and route of BCG administration and consequently the tissue localization have been previously suggested to have a minor impact on the protective immunity against TB [39], [40]. Likewise, in our model, no direct correlation was found between the extents of BCG antigenic load and the magnitudes of BCG-induced T-cell responses or the levels of protection against *Mtb*. In fact, the peak of T-cell responses succeeded early BCG load and waned despite relatively higher BCG titers at week 78.

The BCG-induced T-cell responses in our study were characterized predominantly by IFN-γ and/or TNF-α-producing CD4^+^ T cells and single IFN-γ-positive CD8^+^ T cells. Although the importance of CD4^+^ T cells in protection against *Mtb* has been widely accepted, the role of subunit vaccine-induced CD8^+^ T-cell response has been recently debated [41], [42]. Nonetheless, BCG vaccination-induced CD8^+^ T-cell response has been previously shown to play an important part in the protection against *Mtb* [21], [43], [44]. Multiple studies have shown that IFN-γ and TNF-α cytokines are required for immunity against *Mtb* infection [45], [46]. In contrast, several other studies have shown that the magnitudes of IFN-γ-producing T cells or *in vitro* cytokine levels do not correlate with the degree of protection against TB [34], [40]. Recently, IFN-γ, TNF-α, and IL-2 co-expression by individual polyfunctional CD4^+^ T cells has been proposed as a marker of BCG-induced protective immunity [20]. In our study, the frequency of *Mtb*-specific total or polyfunctional Th1 cytokine-producing CD4^+^ T cells was about two-fold more at the peak (week 32) compared to the expansion phase (week 12), but the levels of protection after *Mtb* challenge was not significantly different. Furthermore, the protection was higher at week 12 compared to week 78, despite comparable magnitudes of BCG-induced T-cell responses. Hence, the correlation between the magnitude of total cytokine-producing T cells or polyfunctional CD4^+^ T cells and the degree of protection against *Mtb* challenge, although positive, appears to be rather weak. Previously, it has been reported that the frequency of BCG-specific CD4^+^, CD8^+^, and γδ T cells and polyfunctional Th1 cytokine-producing CD4^+^ T cells in the blood does not correlate with the protection against TB in BCG-vaccinated children [47]. The increased BCG-burden during the contraction phase and the concurrent decrease in the magnitude and functionality of type-1 cytokine-producing specific T cells is in agreement with previously described negative association between the functional capacity of *Mtb*-specific T cells and *Mtb* load in the sputum of TB patients [48], [49].

It is known that persistent, low-dose antigenic stimulation throughout life may compromise antigen-specific T cells in two ways. First, they may become functionally exhausted and lose essential functional activity that is necessary for immune protection. Second, repeated T-cell stimulation can lead to a loss of the replicative capacity of some antigen-specific T cell populations, as a result of telomere erosion and/or unrepaired DNA damage, a process known as replicative senescence (reviewed in [30]). The induction of senescence in T cells is also known to limit the long-term persistence of immune responses during aging [50]. Therefore, the attrition of specific T-cell responses discerned in our study can be attributed in part to the onset of immune senescence associated with the advancing age in BCG-vaccinated mice. The terminally-differentiated and senescent T cells are characterized by the up-regulation of KLRG-1 expression [51], while functionally exhausted T cells are defined by the early loss of IL-2 production, decreased proliferation, and the increased expression of inhibitory markers like CTLA-4 and PD-1 with low levels of T_CM_ markers CD62L and CCR-7 [27], [52], [53]. Although the terms senescence and exhaustion are often used interchangeably when referring to highly differentiated T cells, available data suggest that both processes are regulated independently of each other [30]. It should be noted that the onset of the contraction of specific T-cell responses in our study was chronicled at the week 52 in BCG-vaccinated mice i.e., at the adult mid-life, and significantly increased expression of KLRG-1 and CTLA-4 was found during the contraction phase on the T cells of BCG-vaccinated mice compared to those of age-matched na?ve mice. In addition, we found comparable magnitudes of specific T-cell cytokine responses in elderly (104-week-old) and younger (8-week-old) mice, 6 weeks post BCG vaccination. Old mice (24-month-old) have been previously shown to effectively control early *Mtb* infection [33], [54], and the BCG vaccination has been found to be equally protective in young, adult and old guinea pigs against TB [55]. Therefore, decreased functional abilities of T cells during the contraction phase of BCG-response observed in our study may not be only due to the advanced-age-related senescence in mice, and it is probable that the chronic persistence of BCG with low grade continuous antigen stimulation throughout life contributes to the attrition of T-cell functions and precipitates immune senescence.

Like *Mtb* infection [25], BCG-induced responses in our study were dominated by CD4^+^ effector/T_EM_ subset secreting IFN-γ and/or TNF-α [56], and the frequency of IL-2^+^ T cells was much lower compared to that reported previously after protective subunit vaccination of mice [57]. Even though CD44^+^CD62L^+^ expression on CD8^+^ T cells in our study suggested an acquisition of the T_CM_ phenotype [58], these cells did not produce IL-2 during the contraction phase and showed concurrent impaired proliferative capacity contrary to the earlier reported characteristics of classical T_CM_ cells [59], [60]. Multiple human and mouse studies have also shown that BCG primarily promotes effector/T_EM_ cells [12], [61]. Many factors may influence development of a long-lived and high-quality memory T cells and of these, antigen load and persistence may be the most important ones [62]. Our study provides a direct evidence for the chronic persistence of BCG load and its correlation with the poor central memory T-cell responses in mice. Previous observations of improved T_CM_ responses after clearance of *Mtb* or BCG in mice and their association with protection provide additional evidence for the correlation between chronic persistence of BCG and poor T_CM_ responses [63]–[65]. Paradoxically, the protective efficacy of BCG in mice also appears to be dependent on the presence of viable bacilli [36] and consequently on the T_EM_ responses. Recently, greater CD4^+^ T_CM_ responses induced by recombinant BCG Δ*ureC*::*hly* vaccine (rBCG) have been shown to be responsible for superior protection compared to canonical BCG against *Mtb* and the T_CM_ responses are shown to coincide with the faster clearance of rBCG in mice [66]. It has been suggested that the canonical BCG is cleared more rapidly in humans than mice and influence the memory phenotype (especially T_CM_ phenotype) of BCG-specific CD4^+^ T cells [15]. However, in this study [15], specific CD4^+^ T cells expressed characteristics of both central memory (CCR7^+^ expression) and effector memory (predominant cytokine IFN-γ and cytotoxic marker expression) following contraction and exhibited a more complex and different phenotype than the classical IL-2^+^T_CM_ cells. The growth and persistence of BCG in humans is not known with certainty, and reports of BCG-lymphadenitis or dissemination years after BCG vaccination are used to support a relatively long life-span of this vaccine in humans [67]–[69]. On the other hand, one would argue that disseminated BCG infections in HIV infected patients and children are not frequent complications or common findings [70]; supporting the viewpoint that viable BCG probably does not persist in children for a long period of time after vaccination. We agree that some aspects of the BCG growth observed in mice such as the resurgence of BCG-burden during the contraction phase of immune response may not necessarily occur in immunocompetent humans and rather this may be a phenomenon seen only in susceptible animal models and children with immune gene deficiency [71]. Like reported observations in mice [7], BCG-induced immunity is thought to wane with time after vaccination at birth in humans and is generally considered to last no more than 10–15 year [72]. However, the time frame for the waning of BCG in humans is still a subject of debate, and a long-term protective efficacy of BCG in some populations has been reported [73]. It is generally agreed, nevertheless, that the waning of BCG-induced protection through childhood and young adult life coincides with the gradual increase in TB incidence in highly TB-endemic regions, which reaches peak in the 25–35-year-old age group [8]. Overall, caution is merited while comparing longitudinal changes in BCG-induced immune responses in mice with those observed in humans.

The persistent antigen load in chronic viral infections and active TB in humans is known to be associated with a progressive impairment of T-cell responses [26], [53], [74], and up-regulation of markers of T-cell dysfunction, terminal differentiation and exhaustion [52], [75], [76]. In our study, BCG-induced response during the contraction phase was characterized by an increased expression of KLRG-1, CTLA-4 and IL-10 production, reduced ability to recall T-cell responses following *Mtb* challenge and loss of protection against *Mtb*. However, we did not observe significant changes in the PD-1 expression on CD4^+^ or CD8^+^ T cells during the three phases of BCG-induced response and our results are at odds with the previous study [77], which documented PD-1 expression on T cells after a high-dose intravenous BCG infection of mice. It has been previously shown that the BCG-vaccinated adults harbor early-stage-differentiated, low PD-1-expressing antigen-specific effector memory CD4^+^ T cells while BCG-vaccinated, latent TB infected (LTBI) adults had late-stage-differentiated PD-1^+^ T cells [78], and the advanced TB patients harbor PD-1^+^ severely exhausted T cells with compromised IFN-γ production [74], [79]. It is tempting to speculate that the resurgence of BCG growth at the late time point (78 weeks) in our study could be due to the lack of immune control and attrition of T-cell functions. However, since BCG CFUs were not enumerated at 104-week time point, this needs further investigation. Overall, our results suggest that the multifactorial functional ability of BCG-induced T cells in-terms of type-1 cytokine production, proliferative and cytotoxic potential and ability to recall immune-mediators post *Mtb* challenge likely correlate with the superior protection against TB. These results also suggest a need to look beyond the quantitative measures of few cytokine markers of vaccine-induced Th1 cells, with improved focus on the combined functional abilities of different immune subsets.

Our results have several implications for the development of effective vaccination strategies using BCG or other live TB vaccines. Although parenteral BCG vaccination via skin was sufficient to induce a durable protective immunity in the lung, our observation of the life-long route-specific compartmentalization of T-cell responses in the draining LNs has important ramifications for the development of effective prime-boost vaccination strategies. The administration of a booster vaccine by a homologous route, involving the same draining LNs as the primer vaccine, may be an effective strategy to induce a superior booster effect in prime-boost scenarios. The ideal time to boost BCG-primed T-cell responses in the mouse model might be after the expansion and peak phase i.e. at week 52 or thereafter. This viewpoint is further supported by a recent data from our laboratory, where boosting with Apa (Rv1860)-based subunit vaccine during the contraction phase offered a stronger boost and imparted significant protection [80]. The data also reinforces the fact that in most published vaccine studies, mice are challenged far too quickly [9] and the longer vaccination-to-challenge intervals, such as 6–8 months after BCG vaccination, will provide better information about the protective efficacy of preclinical vaccines against *Mtb* infection in the mouse model. Further, the loss of protection during the chronic phase of BCG growth and the corresponding increase in KLRG-1^+^ and CTLA-4^+^ T cells highlight the need to develop strategies which can overcome these inhibitory signals and promote a highly distinct or superior quality T-cell responses. One such approach based on our observations could be a rIL-2 therapy to rescue dysfunctional T cells [81]. Recently, it was demonstrated that BCG-mediated protection is lost as *Mtb* infection enters the chronic phase in C57BL/6 mice, but a single boost with H1-subunit vaccine could selectively promote CD4^+^KLRG-1^−^IL-2^+^ T_CM_ cells and impart long-term protection [82], which suggests that strategies to prolong BCG-efficacy without T-cell exhaustion could be pursued.

Overall, our study provided a comprehensive portrait of longitudinal changes in the specific T-cell responses following BCG vaccination in the mouse model. The data suggest an association between the functional capacities of BCG-induced T cells and levels of protection against *Mtb* and provide a rationale for future longitudinal studies optimizing novel prime-boost vaccination strategies and understanding the mechanisms of loss of BCG-induced protection in young adults from the regions of high prevalence of *Mtb* infections. It is a well-established fact that BCG provides the lowest level of protection against TB in the regions with the highest prevalence of latent infection [5], and the caveat is that the certain aspects of the dynamics of BCG-immunity in mice may not inevitably embody human responses. In the light of results presented here, however, it is reasonable to envisage that repeated *Mtb* and environmental mycobacteria exposure and prevalent latent infections in these human populations may further exacerbate T-cell attrition and exhaustion leading to breakdown in BCG-induced anti-TB immunity. Since BCG vaccination-induced T-cell responses mirror those reported by an actual *Mtb* infection in mice and humans, our results indicate that a successful novel TB vaccine should re-educate the immune system rather than reproducing the natural immune responses generated by *Mtb* infection.

## Materials and Methods

### Ethics Statement

All animal experiments performed were in strict accordance with the guidelines of the U.S. Public Health Service Policy on the Humane Care and Use of Animals and the Guide for the Humane Care and Use of Laboratory Animals. All procedures including euthanasia by exsanguination under isoflurane inhalation anesthesia were approved and supervised by the Institutional Animal Care and Use Committee of CDC (Approval numbers SABMOUC1664, 1847, 1490 and SHIMOUC 1422). We obtained 6–8-week-old female BALB/c mice from the Charles River Laboratories. Experimental mice were housed under specific-pathogen-free conditions in the animal biosafety laboratory (ABSL)-II and ABSL-III facilities of the Centers for Disease Control and Prevention (CDC) along with sentinel mice.

### BCG Vaccination and *Mtb* Infection

*M. bovis* BCG Copenhagen vaccine (Danish 1331 strain, Staten Serum Institute, Copenhagen, Denmark) and *Mtb* Erdman challenge strain were provided by the Center for Biologics Evaluation and Research, Food and Drug Administration, Bethesda, MD. For vaccinations of mice, lyophilized BCG was suspended in vaccine diluent (diluted Sauton medium) provided by the supplier. I.n. vaccination was carried out by applying a total of 30 µl of BCG vaccine suspension containing 1×10^6^ CFU drop-wise to the external nares (15 µl per nostril) using a fine tip micropipette and allowing the mouse to inhale the suspension into the lungs naturally [22]. For s.c. vaccination, 50 µl of a BCG suspension was injected above the gluteus superficialis and biceps femoralis muscles of both hind legs using a 26-gauge needle to deliver a total of 1×10^6^ CFU. The vaccine diluent-administered or na?ve mice served as controls.

BCG-vaccinated and control mice were challenged by the airway i.n. route to deliver 5×10^4^ CFU of *Mtb* Erdman [22], [80]. At 48 h following challenge, approximately 0.5% (mean 250 CFU) of the total CFU delivered could be cultured from the lungs. In BCG-vaccinated mice bacillary titers were determined by plating serial dilutions of organ homogenates onto Middlebrook 7H10 agar (BD-Biosciences) supplemented with 0·2% (v/v) glycerol and 10% (v/v) Oleic Albumin Dextrose Catalase (OADC) growth supplement. The BCG growth on plates was confirmed by spoligotyping to rule out atypical mycobacterial co-infection in mice. In *Mtb*-challenged mice, organ CFU were determined on Middlebrook 7H10 agar supplemented with OADC, 10 µg ml^−1^ ampicillin and 50 µg ml^−1^ cycloheximide with or without 2 µg ml^−1^ thiophene 2 carboxylic acid hydrazide (TCH). Mycobacterial colonies were enumerated after 4 weeks of incubation at 37°C.

### ELISPOT assay

Commercially available ELISPOT kits (BD-Biosciences and eBioscience) were used to investigate IFN-γ, IL-2, IL-4 or IL-17A secretion by mouse organ cells according to the manufacturer's protocol as described before [22], [80]. See Text S1 for details.

### Flow cytometry

Mouse organ cells were stained with specific fluorochrome-labeled antibodies to assess the expression of cell surface markers and intracellular cytokines using polychromatic flow cytometry as described earlier [19], [80]. See Text S1 for details.

### ELISA

Serum antibody ELISA was carried out as described previously [83], using 5 µg ml^−1^ of *Mtb* antigen for coating and HRP-conjugated anti-mouse IgG (Sigma-Aldrich), IgG1, IgG2a (BD-Pharmingen) and IgG2b (Santa Cruz Biotech) antibodies for detection.

### CFSE dilution assay

Cells were stained with 5 µM CFSE (Invitrogen) in a total volume of 1 ml for 5 min in the dark [84]. The reaction was stopped using cold PBS containing 5% FBS. The cells were stimulated with or without *Mtb* antigens (1 µg ml^−1^) for 72 h in the presence of 5% CO_2_ at 37°C. The cells were washed, stained with desired anti-mouse fluorochrome-labeled antibodies and acquired using FACS Caliber flow cytometer and analyzed using FlowJo software.

### Cytotoxicity assay

Syngeneic BMDCs were generated using femur bone-marrow cells as described previously [85]. The lung and peritoneal macrophages were obtained after overnight adherence of lung cells or peritoneal lavage of na?ve mice. These antigen presenting cells were pulsed with WCL (10 µg ml^−1^) for 4 h at 37°C in RPMI-1640. Cells were washed (×4) and co-cultured with CD4^+^ or CD8^+^ T cells purified using Dynabeads-untouched-T-cell purification kits (Life Technologies) from the lung or spleen cells of BCG or na?ve mice for 12 h at 37°C. The target and effector cell ratios of 1∶0, 1∶1, 1∶10 and 1∶20 were used. The apoptosis of the target cells after co-culture was assessed using mouse annexin-V staining kit (BD-Biosciences) and polychromatic flow cytometry according to the manufacturer's instructions. In addition to the annexin-V (FITC) and propidium iodide (PI), the cells were stained with antibodies against CD11c (Alexa Fluor-700-clone HL3), CD11b (PE-Cy7-clone M1/70), F4/80 (APC-clone BM8) and CD4 (PerCP-clone RM4-5) or CD8 (PE–clone 53-6.7). The cells were acquired using LSR II flow cytometer and the data was analyzed by Flowjo software.

### In vitro inhibitory receptor blockade and IL-2 treatment

We investigated the potential of rIL-2, anti-KLRG-1 and anti-CTLA-4 antibody treatment to reinvigorate T-cell response in in vitro cultures at the 78 week time point after BCG vaccination of mice. We incubated lung or spleen cells with 10 ng mL^−1^ of rIL-2, with or without WCL stimulation, in ELISPOT and ICS assays. In vitro KLRG-1 or CTLA-4 blockade using corresponding antibodies was also attempted [86], [87]. Different concentrations of KLRG-1 antibody (2F1; eBiosceinces) up to 20 µg mL^−1^ had no detectable effect on the cytokine response in these assays; however, 4F10 CTLA-4 antibody (BD-Biosciences) or purified Fab (20 µg mL^−1^, using Pierce Fab Micro Preparation kit, Thermo Fisher) completely inhibited WCL-specific T-cell cytokine response suggesting inhibitory rather than blocking effect of this antibody [88].

### Statistical analyses

Differences in immune response between groups or time points were assessed by 1-way ANOVA followed by Tukey's post-test and the protective efficacies between vaccinated and control groups were compared by the nonparametric Kruskal-Wallis test followed by the Dunn's post-test (GraphPad Prism program). Unless indicated, all immune response data are presented after subtracting no antigen control values. The *P* value <0.05 was considered significant.

## Supporting Information

Figure S1 **Longitudinal changes in the serum antibody response following BCG vaccination.** BALB/c mice vaccinated with 1×10^6^ CFU BCG by i.n. or s.c. route were euthanized at seven different time points by exsanguination and the serum was isolated from the cardiac blood and evaluated for the antibody response by ELISA. The WCL-specific total IgG (A) or three different IgG subclass (B) levels in the serum of vaccinated or na?ve mice are plotted as an absorbance at 492 nm. The data are mean ± SEM responses of 5–32 individually analyzed mice per time point per group.
(TIF)

Figure S2 **Longitudinal changes in the T-cell responses following BCG vaccination.** (A–B) The lung and spleen cells of i.n. and s.c. BCG-vaccinated mice (*n* = 4/time point/group) were stimulated with *Mtb* STCF, and the magnitudes and polyfunctionality of CD3^+^CD4^+^ and CD3^+^CD8^+^ T cells in-terms of IFN-γ, IL-2 and TNF-α production were determined using polychromatic flow cytometry. The longitudinal changes in the magnitudes of STCF-specific total cytokine producing CD4^+^ (A) or CD8^+^ (B) T cells in the lung (top) and spleen (bottom) are plotted as bar graph while changes in the magnitude of STCF-specific single-, double-, triple-cytokine-producers are plotted as an overlay line graph. The data (A, B) are mean ± s.e.m. responses of 2 (at week 3, 6, 12, 78 and 104) or 3 (at week 32 and 52) independent experiments. *Significantly less total cytokine response was found compared to week 32 using 1-way ANOVA with Tukey's post-test; * *P*<0.05 and ** *P*<0.01. (C) The effect of age and time on the BCG vaccination-induced T-cell response. The magnitudes of total cytokine-producing CD4^+^ T cells in the lungs and spleen after WCL stimulation were investigated at the age and time indicated in three BCG-vaccinated groups (*n* = 4 mice/time point/group). These groups include i) 8-week-old mice vaccinated with BCG and sacrificed 6 weeks later, ii) 8-week-old mice vaccinated with BCG and sacrificed 104 weeks later, and iii) 104-week-old mice vaccinated with BCG and sacrificed 6 weeks later to investigate T-cell response. *Significance in comparison with the group of mice vaccinated at 104 weeks of age and sacrificed 6 weeks later is indicated (1-way ANOVA with Tukey's post-test; * *P*<0.05 ** and *P*<0.01).
(TIF)

Figure S3 **BCG vaccination-induced CD4^+^ and CD8^+^ T cells express cytolytic potentials.** Representative flow cytometry histograms demonstrate annexin-V expression on the cell surface of WCL-pulsed target cells (i.e., CD3^−^F4/80^+^ macrophages) from the (A) lung or (B) peritoneal lavage after co-culture with the purified CD4^+^ (left) or CD8^+^ (right) T cells (at 1∶20 ratio) from the pooled lungs (upper panel) or spleens (lower panel) of BCG-vaccinated or age-matched na?ve mice (*n* = 3/group). (*P<0.05, ** *P*<0.01, using 1-way ANOVA with Tukey's post-test compared to na?ve controls).
(TIF)

Figure S4 **CCR7 (T_CM_ phenotype) expression by T cells following BCG vaccination.** The representative FACS plots demonstrate CCR-7 expression on the unstimulated or WCL-stimulated CD4^+^ and CD8^+^ lung (A), spleen (B) and lymph node T cells at week 12 (top) and week 52 (bottom) following i.n or s.c. BCG vaccination of mice. Data are representative of pooled cells of four mice per time point.
(TIF)

Figure S5 **Longitudinal changes in the CD8^+^ T-cell response of BCG vaccinated and** ***Mtb*** **challenged mice.** (A) The frequency of rESAT-6+rCFP-10-specific total cytokine-positive (of IFN-γ, IL-2 and TNF-α) CD8^+^ T cells from the lung (upper panel) and spleen (lower panel) of BCG-vaccinated and naive mice 6 weeks after challenge at five different time points using ICS. (*P<0.05 and **P<0.01 compared to the corresponding week 32 BCG-vaccinated mice by 1-way ANOVA and Tukey's post-test). At the 32-week time point the rESAT-6+rCFP-10-specific response in the lung of na?ve mice is statistically higher compared to the corresponding vaccinated groups (*P*<0.05). (B) The magnitude of WCL-specific total cytokine-positive CD8^+^ T cells before and 6 weeks after *Mtb* challenge at seven different time points. The data are mean± s.e.m. responses of four mice measured by ICS per time point per group.
(TIF)

Text S1 **Supplementary methods.** Supplemental methods includes details of tissue collection and immune cell isolation, ELISPOT and flow cytometry assay.
(DOCX)
