# Supplementary figures and images for: Attrition of T-Cell Functions and Simultaneous Upregulation of Inhibitory Markers Correspond with the Waning of BCG-Induced Protection against Tuberculosis in Mice
Source: PLoS One. 2014 Nov 24;9(11):e113951. doi: 10.1371/journal.pone.0113951 (PMC4242676; doi:10.1371/journal.pone.0113951)

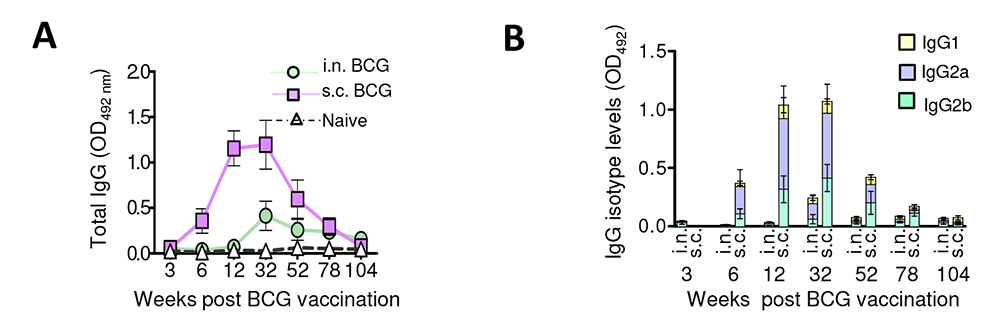

Supplement: Figure S1 — Longitudinal changes in the serum antibody response following BCG vaccination. BALB/c mice vaccinated with 1×10^6^ CFU BCG by i.n. or s.c. route were euthanized at seven different time points by exsanguination and the serum was isolated from the cardiac blood and evaluated for the antibody response by ELISA. The WCL-specific total IgG (A) or three different IgG subclass (B) levels in the serum of vaccinated or na?ve mice are plotted as an absorbance at 492 nm. The data are mean ± SEM responses of 5–32 individually analyzed mice per time point per group. (TIF) [file pone.0113951.s001.tif]

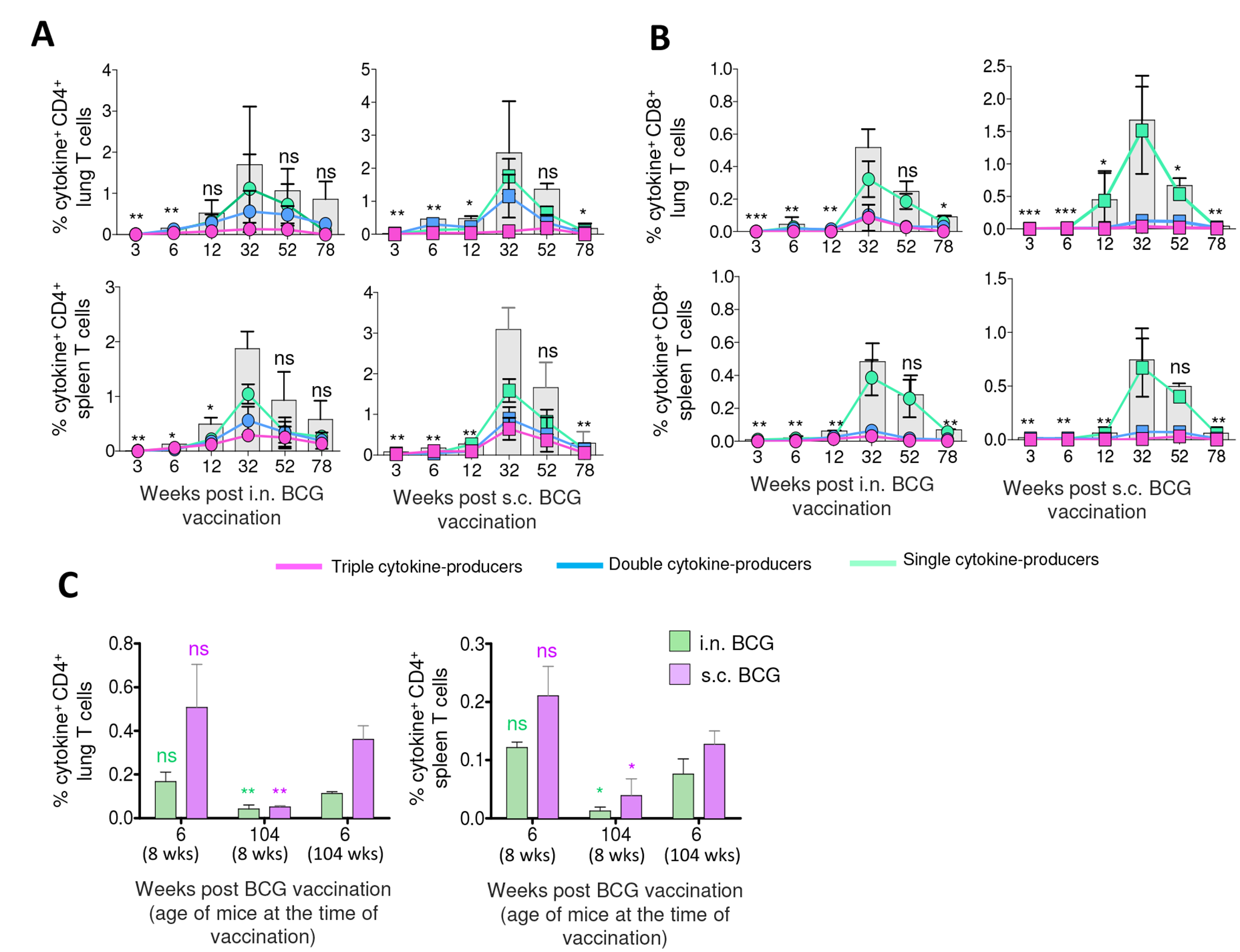

Supplement: Figure S2 — Longitudinal changes in the T-cell responses following BCG vaccination. (A–B) The lung and spleen cells of i.n. and s.c. BCG-vaccinated mice (n = 4/time point/group) were stimulated with Mtb STCF, and the magnitudes and polyfunctionality of CD3+CD4+ and CD3+CD8+ T cells in-terms of IFN-γ, IL-2 and TNF-α production were determined using polychromatic flow cytometry. The longitudinal changes in the magnitudes of STCF-specific total cytokine producing CD4+ (A) or CD8+ (B) T cells in the lung (top) and spleen (bottom) are plotted as bar graph while changes in the magnitude of STCF-specific single-, double-, triple-cytokine-producers are plotted as an overlay line graph. The data (A, B) are mean ± s.e.m. responses of 2 (at week 3, 6, 12, 78 and 104) or 3 (at week 32 and 52) independent experiments. *Significantly less total cytokine response was found compared to week 32 using 1-way ANOVA with Tukey's post-test; * P<0.05 and ** P<0.01. (C) The effect of age and time on the BCG vaccination-induced T-cell response. The magnitudes of total cytokine-producing CD4+ T cells in the lungs and spleen after WCL stimulation were investigated at the age and time indicated in three BCG-vaccinated groups (n = 4 mice/time point/group). These groups include i) 8-week-old mice vaccinated with BCG and sacrificed 6 weeks later, ii) 8-week-old mice vaccinated with BCG and sacrificed 104 weeks later, and iii) 104-week-old mice vaccinated with BCG and sacrificed 6 weeks later to investigate T-cell response. *Significance in comparison with the group of mice vaccinated at 104 weeks of age and sacrificed 6 weeks later is indicated (1-way ANOVA with Tukey's post-test; * P<0.05 ** and P<0.01). (TIF) [file pone.0113951.s002.tif]

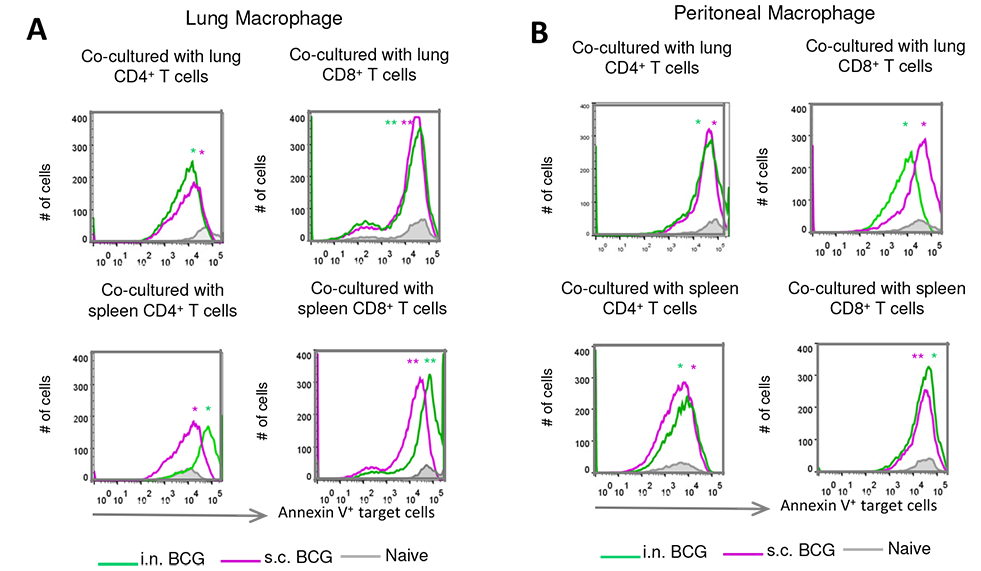

Supplement: Figure S3 — BCG vaccination-induced CD4+ and CD8+ T cells express cytolytic potentials. Representative flow cytometry histograms demonstrate annexin-V expression on the cell surface of WCL-pulsed target cells (i.e., CD3−F4/80+ macrophages) from the (A) lung or (B) peritoneal lavage after co-culture with the purified CD4+ (left) or CD8+ (right) T cells (at 1∶20 ratio) from the pooled lungs (upper panel) or spleens (lower panel) of BCG-vaccinated or age-matched na?ve mice (n = 3/group). (*P<0.05, ** P<0.01, using 1-way ANOVA with Tukey's post-test compared to na?ve controls). (TIF) [file pone.0113951.s003.tif]

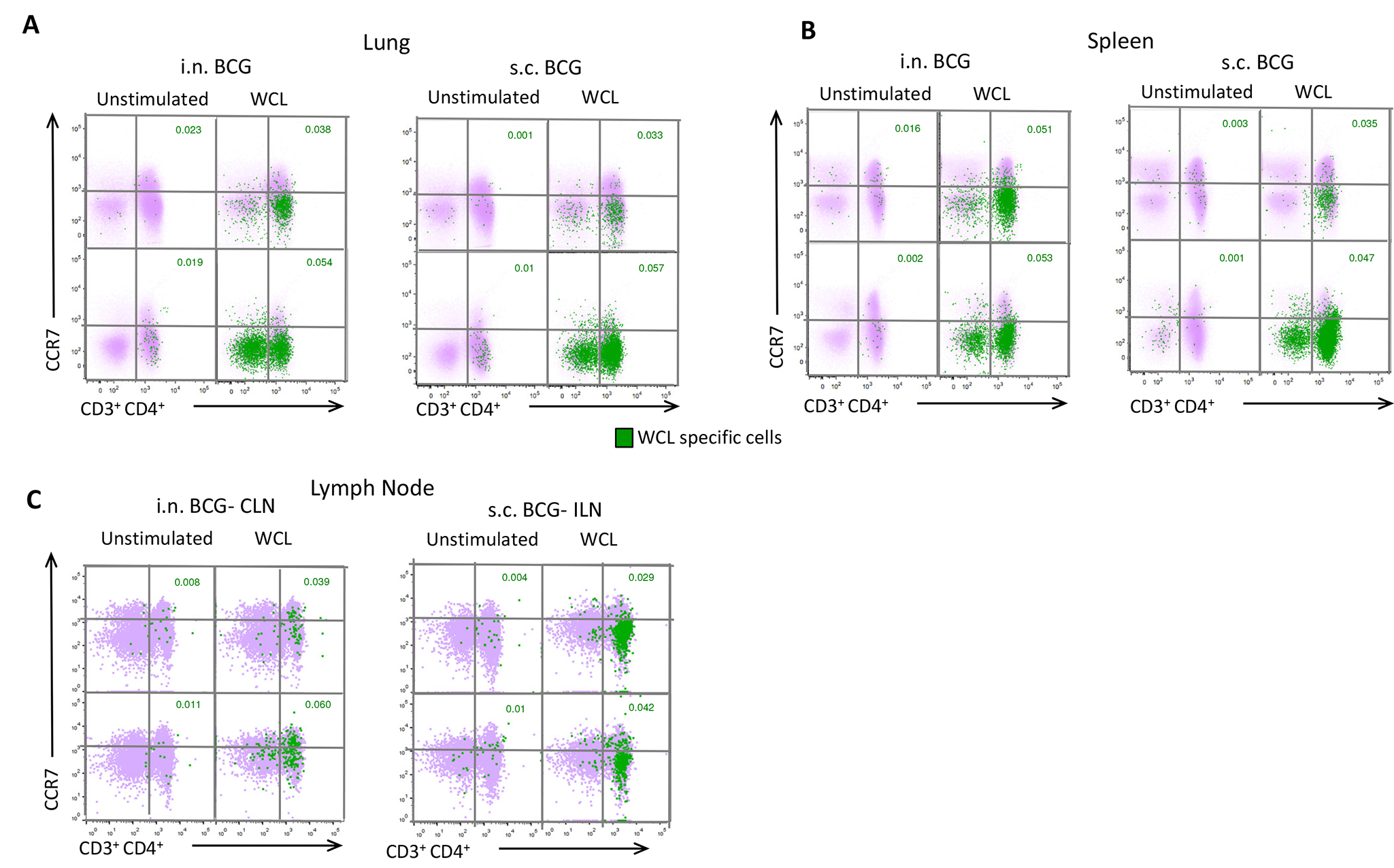

Supplement: Figure S4 — CCR7 (TCM phenotype) expression by T cells following BCG vaccination. The representative FACS plots demonstrate CCR-7 expression on the unstimulated or WCL-stimulated CD4+ and CD8+ lung (A), spleen (B) and lymph node T cells at week 12 (top) and week 52 (bottom) following i.n or s.c. BCG vaccination of mice. Data are representative of pooled cells of four mice per time point. (TIF) [file pone.0113951.s004.tif]

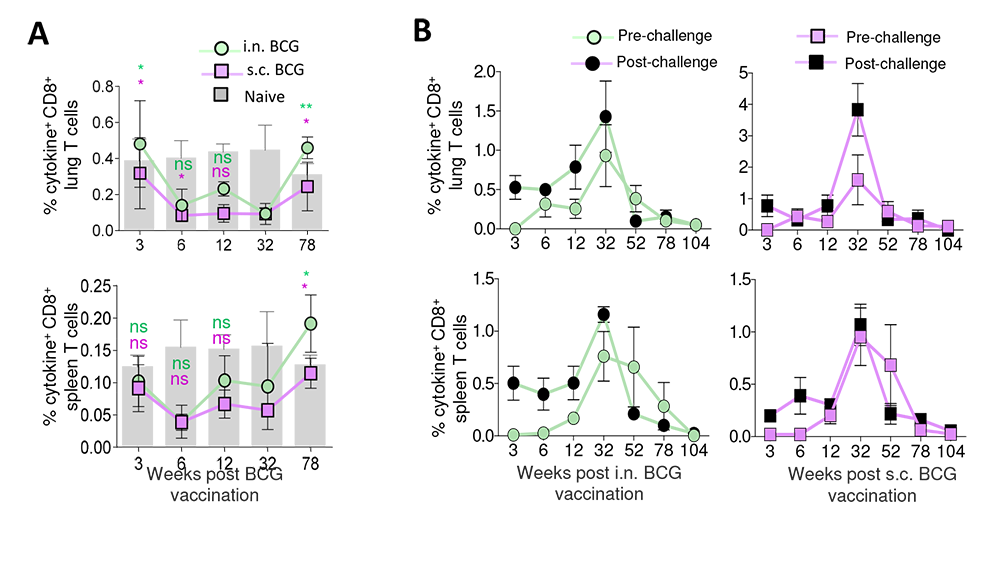

Supplement: Figure S5 — Longitudinal changes in the CD8+ T-cell response of BCG vaccinated and Mtb challenged mice. (A) The frequency of rESAT-6+rCFP-10-specific total cytokine-positive (of IFN-γ, IL-2 and TNF-α) CD8+ T cells from the lung (upper panel) and spleen (lower panel) of BCG-vaccinated and naive mice 6 weeks after challenge at five different time points using ICS. (*P<0.05 and **P<0.01 compared to the corresponding week 32 BCG-vaccinated mice by 1-way ANOVA and Tukey's post-test). At the 32-week time point the rESAT-6+rCFP-10-specific response in the lung of na?ve mice is statistically higher compared to the corresponding vaccinated groups (P<0.05). (B) The magnitude of WCL-specific total cytokine-positive CD8+ T cells before and 6 weeks after Mtb challenge at seven different time points. The data are mean± s.e.m. responses of four mice measured by ICS per time point per group. (TIF) [file pone.0113951.s005.tif]
