# Supplementary material for: Attrition of T-Cell Functions and Simultaneous Upregulation of Inhibitory Markers Correspond with the Waning of BCG-Induced Protection against Tuberculosis in Mice
Source: PLoS One. 2014 Nov 24;9(11):e113951. doi: 10.1371/journal.pone.0113951 (PMC4242676; doi:10.1371/journal.pone.0113951)
Supplement: Text S1 — Supplementary methods. Supplemental methods includes details of tissue collection and immune cell isolation, ELISPOT and flow cytometry assay. (DOCX) [file pone.0113951.s006.docx]

**Attrition of T-Cell Functions and Simultaneous Upregulation of Inhibitory Markers Correspond with the Waning of BCG-Induced Protection against Tuberculosis in Mice**

**Subhadra Nandakumar^1^, Sunil Kannanganat^2^, James E. Posey^1^, Rama Rao Amara^2^ and Suraj B. Sable^1*^**

**^1^**Division of Tuberculosis Elimination, Centers for Disease Control and Prevention, Atlanta, Georgia, USA; ^2^Department of Microbiology and Immunology, Yerkes National Primate Research Center, Emory University, Atlanta, Georgia, USA

**Materials and Methods**

**Collecting Tissues and Isolation of Immune Cells**

The mice were bled by cardiac route and sacrificed by exsanguination at different time points under isoflurane anesthesia. The lungs, spleen, ILNs and superficial CLNs were aseptically removed and organ cells were isolated in RPMI-1640 medium supplemented with 100 IU ml^−1^ penicillin, 50 µg ml^−1^ streptomycin, 1 mM l-glutamine, 25 mM HEPES, 1 mM sodium pyruvate, 5 × 10^−5^ M β-mercaptoethanol, vitamins and nonessential amino acids (Gibco-Invitrogen) and 10% endotoxin-tested heat-inactivated fetal bovine serum (FBS; Atlas Biologicals) as described previously [22]. Briefly, the lungs were digested with an enzyme mixture containing 1 mg ml^−1^ collagenase type IV (Sigma-Aldrich) and 25 U ml^−1^ DNase (Roche) in supplemented RPMI-1640 at 37 °C for 1 h. The digested lung fragments, spleen or LNs were pressed through a 70-µm pore size cell strainer (BD Falcon) to obtain a single cell suspension. The erythrocytes were lysed with RBC lysis buffer (eBioscience) at 22°C, and cells were washed extensively (×4), re-suspended in supplemented RPMI-1640 and counted using an automated cell counter (Countess, Invitrogen) employing the trypan blue dye exclusion method.

**ELISPOT Assay**

We used commercially available ELISPOT kits (BD-Biosciences and eBioscience), and assays were performed according to the manufacturer’s instructions as described before [22, 80]. Briefly, 96-well ELISPOT plates were coated with IFN-γ, IL-2, IL-4 or IL-17A capture antibodies overnight at 4 ºC. Free binding sites were blocked using RPMI-1640 containing 10% FBS for 2 h at room temperature. Cell concentration was adjusted to 1 × 10^6^ cells ml^−1^ for lung and 2 × 10^6^ cells ml^−1^ for spleen and LNs in supplemented RPMI-1640 and added to appropriate wells. Cells were stimulated with either 10 µg ml^−1^ of *Mtb* H37Rv WCL, STCF, rESAT-6, rCFP-10 (National Institute of Health Biodefense and Emerging Infection Research Resources Repository), Concanavalin A (Sigma-Aldrich), or medium alone and incubated for 40 h at 37 ºC in the presence of 5% CO_2_. The plates were washed with PBS containing 0.05% Tween-20 and the cytokine SFU were determined after incubation with the biotin-labeled anti-mouse cytokine detection antibody and horseradish peroxidase-conjugated streptavidin followed by development of the color reaction using 3-amino-9-ethylcarbazole substrate. The number of SFU per well were counted using an automated ELISPOT reader (Cellular Technology Limited).

**Flow Cytometry**

Cells were stained with specific fluorochrome-labeled antibodies to assess the expression of cell surface markers and intracellular cytokines as described earlier [19, 80]. Briefly, 2 × 10^6^ cells were dispensed in 96-well polypropylene plates in supplemented RPMI-1640 medium and stimulated with or without 10 µg ml^-1^ of *Mtb* antigens in a total volume of 200 µl. Stimulation with PMA-ionomycin (1 µg ml^-1^) was used as a positive control. For intracellular cytokine staining (ICS), stimulations were conducted in the presence of antibodies to CD28 and CD49d (1 µg ml^-1^; BD Pharmingen) and the cells were incubated at 37 °C in the presence of 5% CO_2_ for 8 h. Brefeldin A (10 µg ml^-1,^ BD Pharmingen) and monensin (5 µg ml^-1^ Sigma-Aldrich) was added and cells were cultured for additional 4 h. Cells were stained with surface and intracellular anti-mouse antibodies. Anti-mouse fluorochrome-conjugated antibodies used were CD3 (Pacific blue-clone 500A2, phycoerythrin (PE)-clone 500A2, and Allophycocyanin (APC)-clone 145-2C11), CD4 (Alexa Fluor-700-clone RM4-5, PE-clone RM4-5, and Peridinin chlorophyll protein complex (PerCP)-clone RM4-5), CD8 (PerCP-clone 53-6.7 and PE-clone 53-6.7), CD107a/b (Fluorescein isothiocyanate (FITC)-clone ID4B), CD44 (FITC-clone IM7), CD62L (PE-Cy7-clone MEL-14), CTLA-4 (PE-clone UC10-4B9), KLRG-1 (APC-clone 2F1), PD-1 (PE-Cy7-clone RMP1-30 and PE-clone RMP1-30), IFN-γ (APC-clone XMG1.2), TNF-α (PE-Cy7-clone MP6-XT22), IL-2 (PE-clone JES6-5H4) and IL-10 (FITC-clone JES5-16E3). The stained cells were fixed with 1% paraformaldehyde and acquired on the LSR II flow cytometer (BD Immunocytometry Systems) and analyzed using FlowJo software (Tree Star). After subtracting the background values, frequencies of individual or total cytokine secreting CD4^+^ or CD8^+^ T cells were plotted for each antigen.
